# Notch2 and Notch3 Function Together to Regulate Vascular Smooth Muscle Development

**DOI:** 10.1371/journal.pone.0037365

**Published:** 2012-05-17

**Authors:** Qingqing Wang, Ning Zhao, Simone Kennard, Brenda Lilly

**Affiliations:** 1 Center for Cardiovascular and Pulmonary Research, Nationwide Children's Hospital, Columbus, Ohio, United States of America; 2 Department of Pediatrics, The Ohio State University, Columbus, Ohio, United States of America; 3 Vascular Biology Center, Medical College of Georgia, Augusta, Georgia, United States of America; William Harvey Research Institute, Barts and The London School of Medicine and Dentistry, Queen Mary University of London, United Kingdom

## Abstract

Notch signaling has been implicated in the regulation of smooth muscle differentiation, but the precise role of Notch receptors is ill defined. Although Notch3 receptor expression is high in smooth muscle, Notch3 mutant mice are viable and display only mild defects in vascular patterning and smooth muscle differentiation. Notch2 is also expressed in smooth muscle and Notch2 mutant mice show cardiovascular abnormalities indicative of smooth muscle defects. Together, these findings infer that Notch2 and Notch3 act together to govern vascular development and smooth muscle differentiation. To address this hypothesis, we characterized the phenotype of mice with a combined deficiency in Notch2 and Notch3. Our results show that when Notch2 and Notch3 genes are simultaneously disrupted, mice die in utero at mid-gestation due to severe vascular abnormalities. Assembly of the vascular network occurs normally as assessed by Pecam1 expression, however smooth muscle cells surrounding the vessels are grossly deficient leading to vascular collapse. In vitro analysis show that both Notch2 and Notch3 robustly activate smooth muscle differentiation genes, and Notch3, but not Notch2 is a target of Notch signaling. These data highlight the combined actions of the Notch receptors in the regulation of vascular development, and suggest that while these receptors exhibit compensatory roles in smooth muscle, their functions are not entirely overlapping.

## Introduction

The Notch family of receptors is evolutionarily conserved and critical for cell fate determination and differentiation [1], [2]. Each of the four Notch receptors present in mammals (Notch 1–4) is activated by a membrane-bound ligand (Jagged-1,2/Delta-like-1,3,4), which promotes receptor cleavage releasing a Notch intracellular domain (NICD) that translocates to the nucleus. In the nucleus, the NICD binds to the transcription factor CSL (CBF-1/RBP-Jκ, Su(H), and Lag-1) and regulates downstream gene expression such as Hes (hairy/enhancer of split) and Hey (Hairy-related, also referred to as Hrt, CHF, HESR) family members [3]. In the vasculature, Notch activation regulates the expression of angiogenic factors, including members of the vascular endothelial growth factor (VEGF) pathway [4], and platelet-derived growth factor receptor-ß [5]. Not surprisingly, functional studies have demonstrated a role for Notch signaling in angiogenic remodeling, arterial/venous specification, and in endothelial tip cell differentiation [6], [7]. While, many of these studies focused on the actions of Notch signaling in the endothelium, others have identified a role for Notch activation in vascular smooth muscle development [8]. One report showed that expression of the Notch ligand Jagged1 (Jag1) on endothelial cells is essential for neighboring vascular smooth muscle differentiation [9], indicating a requirement for Notch receptors on smooth muscle cells. The Notch3 receptor is highly enriched in smooth muscle [10], [11] and Notch3 knockout mice, while viable, display vascular smooth muscle defects associated with postnatal maturation and arterial specification [12], [13]. We previously demonstrated that differentiation of vascular smooth muscle cells by endothelial cells was dependent upon NOTCH3 [14]. Notch2 is more widely expressed than Notch3, but is also present in vascular smooth muscle cells [11], [15], [16]. Two studies in particular have hinted at a role in smooth muscle cell regulation. McCright et al., reported that Notch2 hypomorphic knockout mice exhibit widespread hemorrhaging mid to late gestation [17]. Whereas, a neural crest-specific deletion of Notch2 causes an underdeveloped outflow tract due to decreased smooth muscle [15]. Thus, given the collective evidence for a role of the Notch receptors in regulating smooth muscle differentiation, we hypothesized that multiple Notch receptors, particularly Notch2 and Notch3 act together to regulate vascular smooth muscle differentiation. Here, we perform a phenotypic analysis of mice deficient in the Notch2 and Notch3 genes. Our results show that together these genes regulate vascular smooth muscle development. Mice deficient in both genes die during mid-gestation from severe vascular defects associated with an absence of differentiated smooth muscle cells. These data are the first to demonstrate a critical role for these Notch receptors in vascular development, and highlight the combined role they play in regulating smooth muscle differentiation.

## Results

### Combined mutations in Notch2 and Notch3 genes cause embryonic lethality

Prior analysis showed that Notch3 null mice are viable and fertile with minor, yet significant postnatal defects in smooth muscle structure and vascular function [12], [13], [18]. Because Notch2 is also expressed in smooth muscle and a neural crest-specific Notch2 mutation causes vascular patterning defects [11], [15], [16], we set out to determine the phenotype of mice with a combined deficiency in the Notch2 and Notch3 genes. We crossed previously generated Notch2 hypomorph [17] and Notch3 null [19] mice to create double heterozygotes *(Notch2^−/+^;Notch3^−/+^)*, which we intercrossed to generate the genotypes for comparison. From a double heterozygous cross, nine different genotypes were produced. Initial analysis indicated that the *Notch2^−/−^;Notch3^−/−^* embryos did not survive to embryonic day (E)12.5 of gestation (Table S1). Double mutant embryos were recovered at expected Mendelian ratios at this time point, however all *Notch2^−/−^;Notch3^−/−^* embryos were small, pale (bloodless) and were being resorbed. At E11.5 the *Notch2^−/−^;Notch3^−/−^* embryos were easily distinguishable from wildtype and heterozygous littermates with obvious signs of hemorrhaging and yolk sac defects. This primary analysis indicated that the combined loss of Notch2 and Notch3 resulted in embryonic lethality around E11.5; thus we focused on this time point and those just prior to determine the cause of lethality.

**Figure 1:**
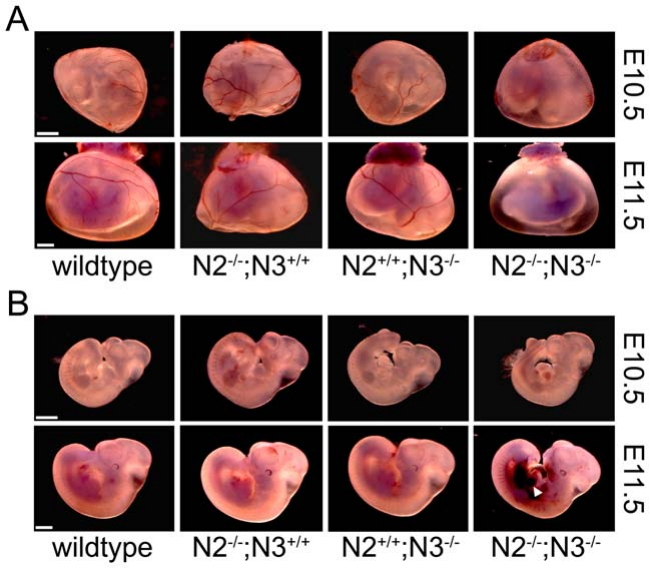
Combined mutations in Notch2 and Notch3 cause defects in vascular development. Yolk sacs (A) and embryos (B) at E10.5 and E11.5 were dissected and photographed with a stereo microscope. At E10.5, *Notch2^−/−^;Notch3^−/−^ (N2^−/−^;N3^−/−^)* embryos exhibit a decrease in yolk sac blood vessels, while the embryo proper is relatively normal in appearance. At E11.5, *Notch2^−/−^;Notch3^−/−^* mice show severe vascular defects in both yolk sac and embryo. Yolk sac blood vessels are not visible and extensive hemorrhaging is seen in the embryo (arrowhead). Blood vessels are grossly normal in *Notch2^−/−^ (N2^−/−^;N3^+/+^)* and *Notch3^−/−^ (N2^+/+^;N3^−/−^)* embryos. Scale bar = 1 mm.

**Figure 2:**
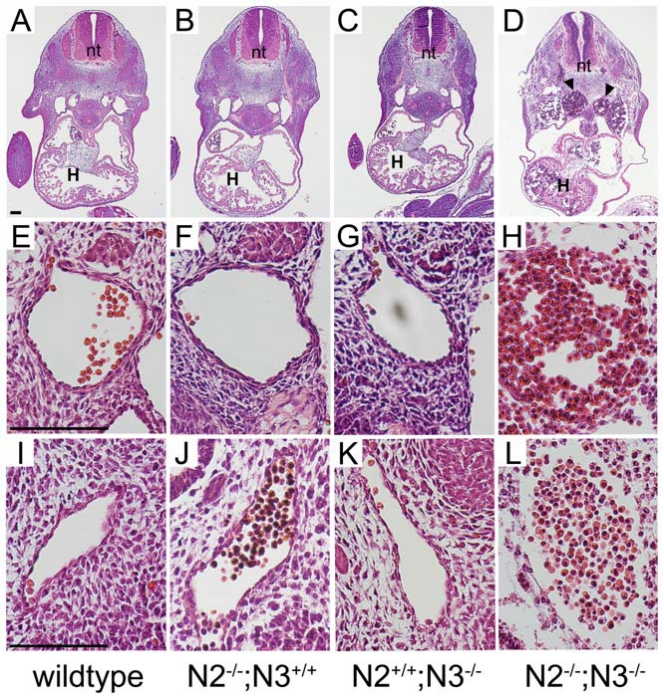
Embryos lacking both Notch2 and Notch3 have disrupted blood vessels. Hematoxylin and eosin staining of transverse sections of E11.5 embryos through the heart and midsection (A–D), descending aorta (E–H), and caudal aorta (I–L). In the *Notch2^−/−^;Notch3^−/−^ (N2^−/−^;N3^−/−^)* embryos, the paired dorsal aorta is expanded in size and filled with blood (D, arrowheads). Higher magnification of blood vessels in double mutant embryos show a lack of cells surrounding the lumen (H, L). The overall structure of blood vessels appears relatively normal in the single *Notch2^−/−^ (N2^−/−^;N3^+/+^)* and *Notch3^−/−^ (N2^+/+^;N3^−/−^)* mice. Scale bar = 100 µm. (H) heart, (nt) neural tube.

For simplicity, we chose to perform a comparative examination of wildtype embryos with the three genotypes harboring homozygous mutant alleles alone and in combination *(Notch2^−/−^;Notch3^+/+^, Notch2^+/+^;Notch3^−/−^, Notch2^−/−^;Notch3^−/−^)*. Gross analysis of the yolk sacs by light microscopy revealed normal vascular patterning in wildtype and single mutant embryos at E10.5, however 45% of the double mutant yolk sacs had a decrease in visible blood vessels (Figure 1A and Table 1). At E11.5, 92% of double mutant embryos had reduced blood vessels in the yolk sacs (Figure 1A, and Table 1), while only a small percentage of the Notch2 and Notch3 mice had observable vascular defects within their yolk sacs (Table 1). Examination of the embryo proper at E10.5 showed that all genotypes were phenotypically normal and appeared similar in size with no apparent structural defects (Figure 1B). At E11.5, double mutant embryos had a combination of abdominal and cerebral hemorrhaging, pericardial effusion, and in some cases were smaller in size (Figure 1B). The *Notch3^−/−^* mice were indistinguishable from the wildtype embryos and a small percentage of *Notch2^−/−^* mice exhibited visible hemorrhaging, consistent with previous findings [17]. These data suggested that the primary defect of the mutant embryos was vascular insufficiency leading to the demise of the embryos. To examine potential structural defects of blood vessels, embryos were cross-sectioned and stained with hematoxylin and eosin (H&E). At E10.5, the overall structure of the blood vessels from the four different genotypes were indistinguishable, with all having intact vessel walls (Figure S1). At E11.5, embryos deficient in both Notch2 and Notch3 had severely compromised blood vessel structure (Figure 2). Vessels were enlarged and filled with blood, suggesting improper flow, while the vessel walls were thin and in certain cases appeared to incompletely enclose the lumen. The defect was observed in the dorsal aorta and caudal aorta (Figure 2), and was also apparent in smaller arteries like the carotid arteries (not shown). In contrast, the overall structure of the blood vessels in the *Notch2^−/−^* and *Notch3^−/−^* embryos at E11.5 was similar to wildtype.

**Table 1:** Notch2 (N2) and Notch3 (N3) mutant embryos with yolk sac defects.

	Genotypes				
Age		N2^+/+^;N3^+/+^	N2^−/−^;N3^+/+^	N2^+/+^;N3^−/−^	N2^−/−^;N3^−/−^
E10.5	%	0%	0%	0%	45%
	#	0 of 14	0 of 17	0 of 18	10 of 22
E11.5	%	0%	11%	8%	92%
	#	0 of 16	2 of 19	1 of 13	12 of 13

### Notch2 and Notch3 double mutants show deficiencies in smooth muscle cells

**Figure 3:**
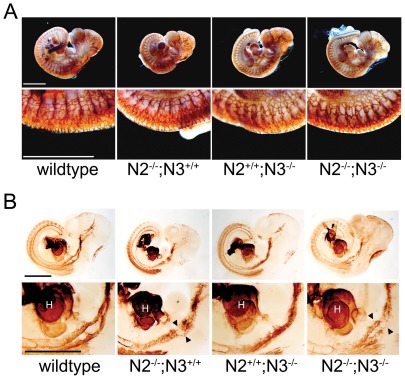
Notch2^−/−^ and Notch2^−/−^;Notch3^−/−^ embryos develop a normal vascular plexus but have disrupted vascular smooth muscle cells. Whole-mount embryos at E10.5 were stained for Pecam1 (A) or SMA (B). The vascular plexus is well formed in all mutant embryos with normal vessel patterning seen in large vessels (A, upper panels) and smaller intersomitic vessels (A, lower panels). Whole-mount immunostaining for SMA demonstrates less SMA-positive cells in the dorsal aorta of *Notch2^−/−^ (N2^−/−^;N3^+/+^)* and *Notch2^−/−^;Notch3^−/−^ (N2^−/−^;N3^−/−^)* (B, lower panels) embryos compared to wildtype and *Notch3^−/−^ (N2^+/+^;N3^−/−^)* embryos. Arrowheads point to paired dorsal aorta. Scale bar = 1 mm. (H) heart.

**Figure 4:**
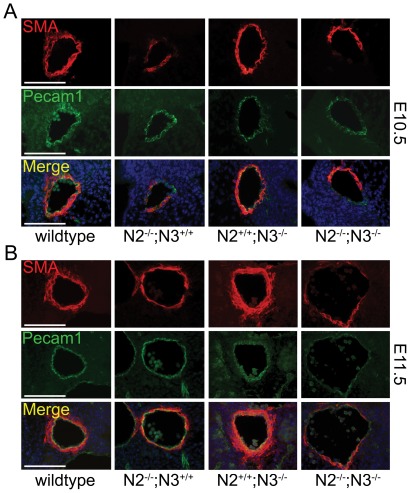
Notch2 and Notch2/Notch3 double mutant embryos exhibit diminished smooth muscle cell marker expression. Transverse sections of embryos at E10.5 (A) and E11.5 (B) were stained for SMA (red) and Pecam1 (green). *Notch2^−/−^ (N2^−/−^;N3^+/+^)* and *Notch2^−/−^;Notch3^−/−^ (N2^−/−^;N3^−/−^)* embryos exhibit less SMA-positive cells in the dorsal aorta at E10.5 compared to wild-type and *Notch3^−/−^ (N2^+/+^;N3^−/−^)* embryos, while Pecam1 levels are similar in all genotypes. At E11.5, *Notch2^−/−^;Notch3^−/−^* embryos show an even greater loss of SMA expression, with increased vessel diameter. Merged images also show DAPI stain (blue) to highlight nuclei. Scale bar = 100 µm.

We stained whole embryos with the endothelial cell marker Pecam1 and the smooth muscle marker smooth muscle α-actin (SMA) to assess the overall structure of the blood vessels in mutant embryos. At E10.5, Pecam1 staining in the double mutant embryos appeared similar to wildtype and the single mutant embryos, indicating that vascular patterning was grossly normal and endothelial cells were being properly positioned (Figure 3A). Staining with SMA showed similar expression levels in the heart of all genotypes, but the *Notch2^−/−^;Notch3^−/−^* embryos and *Notch2^−/−^* embryos exhibited a decrease in the SMA staining within the paired dorsal aorta (Figure 3B). These data indicated that the loss of Notch2 and the combined loss of Notch2 and Notch3 result in a decrease in smooth muscle cells surrounding the blood vessel walls. Section staining through blood vessels at E10.5 and E11.5 showed a consistent result with the whole mount analysis. At E10.5, Pecam1 expression showed a similar patterning in all genotypes, while SMA expression was greatly reduced in both the *Notch2^−/−^* and *Notch2^−/−^;Notch3^−/−^* embryos (Figure 4A, Figure S2). At this stage SMA expression appeared mottled, with many vessels exhibiting SMA staining that was asymmetrically localized. At E11.5, the expression pattern of Pecam1 was decreased in the double mutant embryos, with SMA expression barely detectable (Figure 4B, Figure S2). Notch2 mutant mice had reduced Pecam1 staining compared to wildype and *Notch3^−/−^* embryos, and showed SMA staining that was moderately reduced from wildtype (Figure 4B, Figure S2). Collectively, our results show that at E10.5, vessels from *Notch2^−/−^* and *Notch2^−/−^;Notch3^−/−^* embryos are similar, however at E11.5, the loss of both genes causes a dramatic reduction in SMA staining and compromised vessel structure. These defects were exclusive to smooth muscle of Notch2/Notch3 mutant embryos, as expression of SMA in the myocardium was comparable to wildtype (Figure S3).

### Combined Notch2 and Notch3 mutations cause yolk sac defects

**Figure 5:**
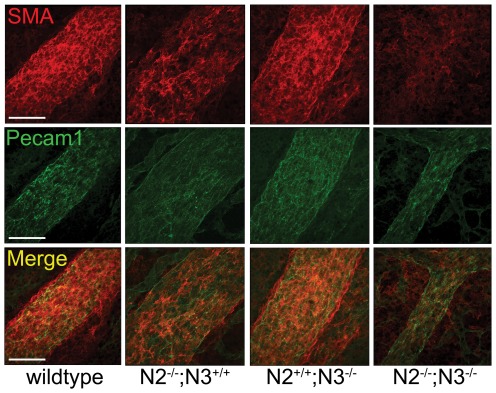
SMA expression is decreased in yolk sacs of Notch2 and Notch2/Notch3 double mutant embryos. Yolk sacs collected at E11.5 were stained for SMA (red) and Pecam1 (green). SMA is prominently expressed around the blood vessels in yolk sacs of wildtype and *Notch3^−/−^ (N2^+/+^;N3^−/−^)* embryos, whereas its expression is significantly decreased in *Notch2^−/−^ (N2^−/−^;N3^+/+^)* and *Notch2^−/−^;Notch3^−/−^ (N2^−/−^;N3^−/−^)* vessels. Yolk sacs of the *Notch2^−/−^;Notch3^−/−^* embryos have blood vessels that appear structurally deficient compared to the other genotypes. Scale bar = 100 µm.

**Figure 6:**
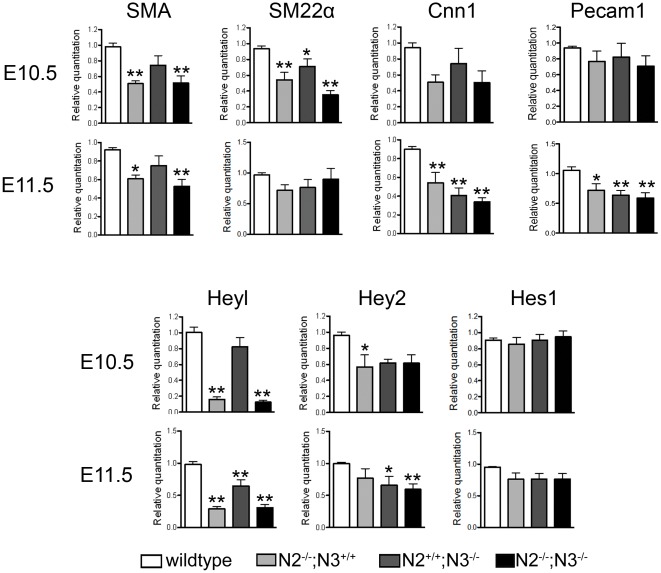
Smooth muscle differentiation and Notch target genes are down regulated in Notch2 and Notch2/Notch3 double mutants. Gene expression analysis (qPCR) using yolk sac RNA demonstrates a reduction of smooth muscle-specific genes SMA, SM22α, and Cnn1 in *Notch2^−/−^ (N2^−/−^;N3^+/+^)* and *Notch2^−/−^;Notch3^−/−^ (N2^−/−^;N3^−/−^)*. Notch signaling target gene Heyl shows a robust decrease at E10.5 and E11.5 in the *Notch2^−/−^ (N2^−/−^;N3^+/+^)* and *Notch2^−/−^;Notch3^−/−^ (N2^−/−^;N3^−/−^)* yolk sacs, while Hey2 shows a slight decrease and Hes1 expression is unaffected in the mutant genotypes. Data represent relative mRNA expression levels normalized to 18S rRNA. * *P*<0.05, ** *P*<0.01 compared to wildtype.

Our initial analysis showed that the yolk sac vasculature is disrupted in double mutant embryos, so we additionally analyzed these blood vessels to determine if similar defects existed in this vascularized tissue. Yolk sac blood vessels at E11.5 had slightly reduced amounts of Pecam1 staining in both the *Notch2^−/−^* and *Notch2^−/−^;Notch3^−/−^* genotypes and reduced SMA expression (Figure 5). Similar to the embryos, the yolk sac vessels of the double mutant mice appeared structurally fragile, and in many instances were collapsed. Because yolk sacs are a highly vascularized tissue, we used this tissue to assess gene expression of smooth muscle markers and Notch signaling targets by qPCR (Figure 6). SMA RNA levels showed a decrease consistent with immunofluorescence staining, and another early marker of smooth muscle cells, SM22α exhibited a decrease at E10.5 but not at E11.5. The pronounced decrease observed at E10.5 compared to E11.5 likely reflects continued expression of these markers in nonvascular cells within the whole yolk sac. A later smooth muscle marker gene, Calponin-h1 (Cnn1) exhibited decreased transcripts in all mutant genotypes at E11.5, but not E10.5. Consistent with our immunohistochemistry results, Pecam1 expression was normal at E10.5, and showed a reduction at E11.5 in all mutant genotypes. Examination of Notch target genes indicated no significant difference in Hes1 expression and a small decrease in Hey2. Heyl expression was dramatically affected by the loss of Notch2 and the combined Notch2/Notch3 mutant, but only slightly affected by the loss of Notch3.

### Notch2 and Notch3 activate downstream targets with similar efficiency

**Figure 7:**
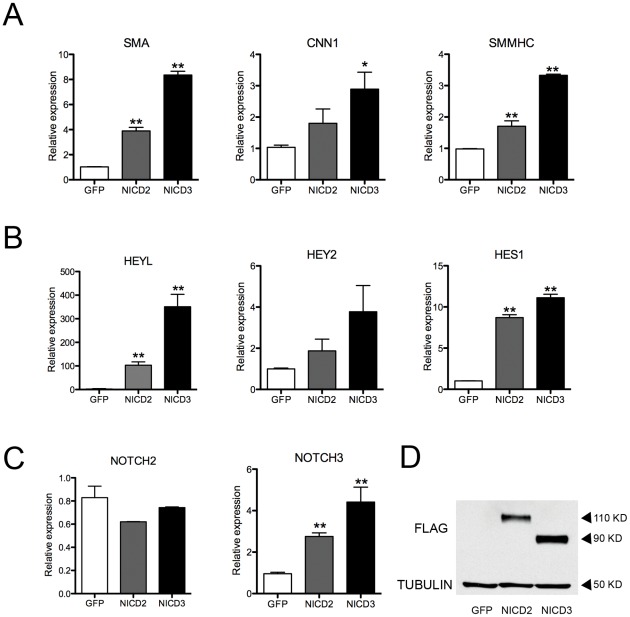
NICD2 and NICD3 can directly activate Notch targets and smooth muscle marker genes. Activated forms of Notch2 (NICD2) and Notch3 (NICD3) were introduced into human aortic smooth muscle cells by lentiviral transduction, followed by qPCR to analyze gene expression. Both NICD2 and NICD3 robustly activate expression of smooth muscle genes, SMA, CNN1, and SM-MHC (A), and also activate Notch targets HEYL, HEY2, and HES1 (B), compared to a GFP-expressing control. Both NICD2 and NICD3 activate endogenous NOTCH3 expression, but not NOTCH2 expression (C). Western blot demonstrates expression of the NICD2 and NICD3 constructs with a FLAG antibody (D). Data represent relative mRNA expression levels normalized to 18S rRNA. * *P*<0.05, ** *P*<0.01 compared to GFP control.

Because our results suggested that both Notch2 and Notch3 contribute to smooth muscle differentiation we wanted to determine if they could directly activate smooth muscle-specific gene expression. We overexpressed the intracellular domains of the Notch2 (NICD2) and Notch3 (NICD3) by lentiviral infection of human aortic smooth muscle cells and examined gene expression by qPCR. Both NICD2 and NICD3 activated the expression of smooth muscle differentiation genes SMA, CNN1 and smooth muscle myosin heavy chain (SM-MHC) (Figure 7A). Similarly, overexpression of NICD2 and NICD3 activated known Notch target genes (Figure 7B). Previously we showed that NOTCH3 was induced in smooth muscle by Notch signaling and could activate its own transcription through autoregulation [14]. Consistent with this, NICD3 activated transcription of the endogenous NOTCH3 gene, but was not able to induce the expression of NOTCH2 in aortic smooth muscle cells. NICD2 could also promote the expression of NOTCH3, but could not induce the expression of endogenous NOTCH2 transcripts (Figure 7C). Overall, our data demonstrate a critical role for Notch signaling in the development of vascular smooth muscle cell, and indicate that Notch2 and Notch3 have overlapping yet distinct roles in governing smooth muscle differentiation.

## Discussion

Although several lines of evidence have implicated Notch signaling in vascular smooth muscle development, the data supporting this notion has been less conclusive. Here we show that combined mutations of Notch2 and Notch3 genes in mice results in severe cardiovascular defects with the underlying cause due to a lack of smooth muscle differentiation. Previous studies on Notch receptor function in vascular smooth muscle cells have largely focused on Notch3, due to its relatively specific expression within this cell type and the known association with the human cerebral vascular disease, cerebral autosomal dominant arteriopathy with subcortical infarcts and leukoencephalopathy (CADASIL) [10], [11], [20]. Data from knockout mice indicated that loss of Notch3 results in smooth muscle maturation defects and causes deficiencies in arterial specification [12]. Further studies with postnatal mice revealed a role for Notch3 in the regulation of proliferation and vascular tone [18], [21]; however, Notch3 null mice undergo a normal restenosis response following vascular injury [22]. These data clearly indicate that Notch3 regulates aspects of smooth muscle function, but is non-essential for smooth muscle differentiation. In vitro data from our lab indicated that Notch3 drives expression of smooth muscle genes, and in a model of endothelial cell-induced smooth muscle differentiation, Notch3 is critical for smooth muscle gene expression [14].

While previous data showed that other Notch receptors do not compensate in the absence of Notch3 through increased expression [11], their presence in smooth muscle cells may contribute to smooth muscle differentiation. Indeed, Notch2 has been shown to be strongly expressed in smooth muscle and data from knockout mice show cardiac outflow tract anomalies consistent with smooth muscle defects [15], [17]. Because Notch2 null mice die very early due to massive cell death [16], in this study we utilized a hypomorphic allele, which had been previously characterized to have cardiovascular defects [17]. However the smooth muscle differentiation profile of these hypomorphic mice had not been reported. In our analysis we show for the first time, that Notch2 hyopmorphic mice have a loss of smooth muscle markers as early as E10.5, which likely leads to later defects associated with hemorrhage and outflow tract defects. In contrast, Notch3 null mice at E10.5 exhibit normal vascular structure, with no apparent signs of defects. Loss of function of the two Notch genes gives rise to a complete breakdown of the vascular wall at E11.5. At E10.5, the expression of SMA looks comparable in the Notch2 and double mutant embryos, suggesting that Notch2 activity predominates at this early stage, however at E11.5, the double mutant vessels appear to lose most of their smooth muscle cells, while the Notch2 embryos retain the expression of smooth muscle marker, SMA. Importantly, the double mutant embryos still express some SMA and SM22α, implying that these Notch receptors are not completely necessary for the expression of these smooth muscle marker genes during differentiation. Additional signaling pathways are likely involved in maintaining expression during differentiation, such as those driven by TGFß or serum response factor (SRF) [23].

Previously it was shown that endothelial cell-expressed Notch ligand Jagged1 is critical for smooth muscle differentiation [9]. Our previous data and findings shown here, indicate that Notch3 expression, but not Notch2 is induced by Notch signaling [14]. Possibly, within smooth muscle cells Notch2 is activated by the Jagged1 ligand on neighboring endothelial cells to establish the first wave of Notch activation and differentiation. During this phase, Notch2 activates not only smooth muscle genes, but also Notch3 expression and together they drive the differentiation program. In the absence of Notch2, smooth muscle differentiation is delayed slightly, however the presence of Notch3 can maintain vascular integrity. In the absence of Notch3, Notch2 expression is sufficient for the initiation of smooth muscle differentiation, but is not capable of the fine-tuning required for the later steps of maturation. In the absence of both genes, smooth muscle cells cannot differentiate properly, the vessels carrying blood through the embryos fail, resulting in hemorrhaging and eventual collapse of the vessel. Overall, our data show for the first time a critical role for Notch receptors in vascular smooth muscle development, and suggest that propagation of Notch signaling in this cell type requires the combined efforts of Notch2 and Notch3. The actual mechanisms by which Notch2 and Notch3 coordinate to regulate vascular development are not known. Differences in how they are regulated by upstream transcription factors might contribute to individual temporal functions that converge within blood vessels. Alternatively, unique structural features of each Notch family member could contribute to distinct downstream interactions and responses that when acting together shape the vascular landscape.

## Materials and Methods

### Ethics statement

All mouse studies were carried out in accordance with protocols approved by the Institutional Animal Care and Use Committee (IACUC) at the Research Institute at Nationwide Children's Hospital. The Nationwide Children's Hospital Research Institute IACUC specifically approved this study.

### Mouse lines, genotyping and crosses

All strains were maintained in C57Bl/6 background. *Notch2^del1^* [17] and *Notch3^dl^* [19] single mutant mice were generated and generously provided by Dr. Thomas Gridley. *Notch2^del1/+^*, referred to here as *Notch2^−/+^ (N2^−/+^)* mice were crossed with *Notch3^dl/dl^*, referred to here as *Notch3^−/−^ (N3^−/−^)* mice to generate *Notch2^−/+^;Notch3^−/+^* double heterozygous mice. To produce embryos for analysis, *Notch2^−/+^;Notch3^−/+^* mice were intercrossed. Nine different genotypes of embryos were generated and collected as shown in Table 1. Their numbers were tested for goodness of fit to expected Mendelian segregation. Embryos were considered embryonic (E) day 0.5 (E0.5) at the day when vaginal plug was observed. Genotyping of mice and embryos was carried out by PCR with Notch2wtsp3: 5′-CCA GTG TGC CAC AGG TAA GTG-3′, Notch2wtsp4: 5′-TCT CCA TAT TGA TGA GCC ATG C-3′, Notch2dlsp6: 5′ –TTC CTG ACT AGG GGA GGA GTA G-3′. Notch3wt1: 5′-CCA TGA GGA TGC TAT CTG TGA C-3′, Notch3wt2: 5′-CAC ATT GGC ACA AGA ATG AGC C-3′, Notch3dl1: 5′-GGT ACT GAG AAC CAA ACT CAG C-3′, Notch3dl2: 5′-TCG CCT TCT ATC GCC TTC TTG A-3′.

### Quantitative RT-PCR (qPCR)

Total RNA was extracted using RNeasy Mini Kit (QIAGEN, Cat: 74104) from a minimum of five yolk sacs corresponding to each genotype. RNA from cultured cells was isolated by TRIzol (Invitrogen). Reverse transcription was performed using M-MLV reverse transcriptase (Invitrogen, Cat: 28025-013). SYBR green detection of PCR amplicons was performed using an ABI qPCR machine. Corresponding gene expression level was normalized to 18S rRNA or Gapdh from the same sample. Primer sequences are listed in Table S2.

### Whole-mount immunohistochemistry

Embryos or yolk sacs were harvested in cold phosphate buffered saline (PBS) and fixed in 4% paraformaldehyde overnight at 4°C or 1 hour at room temperature. For embryo staining, the embryos were dehydrated in a graded methanol series, and bleached with Methanol: DMSO: 3% H_2_O_2_ (4∶1∶1) or PBS: 3% H_2_O_2_ (4∶1) to block endogenous peroxidase. Embryos were then blocked and permeabilized with 5% instant nonfat milk or goat serum and 0.3% Triton-X-100. Primary antibodies, Smooth muscle α-actin (SMA) (1∶500, SIGMA, Cat: A2547) or Pecam1 (1∶50, BD Pharmingen, Cat: 550274) were incubated overnight at 4°C, followed by incubation with appropriate HRP-conjugated secondary antibody (1∶500) overnight at 4°C. The color reaction was done in PBT (PBS+0.1% tween-20) containing 0.5 mg/ml 3,3′-diaminobenzidine (DAB, SIGMA, Cat: D-5673) and 0.01% H_2_O_2_. Embryos were dehydrated and cleared in benzyl alcohol: benzyl benzoate (1∶2) (SIGMA). Pictures were captured with a dissecting microscope (Leica, M156C). Immunostaining of whole yolk sacs was performed as previously described with modifications [13]. In brief, after fixation, yolk sacs were placed in cold methanol for 10 minutes, blocked in 5% goat serum and 0.3% Triton-X-100 for 1 hour at room temperature, followed by Pecam1 and SMA antibody incubation for 2 hours at 37°C. Yolk sacs were then incubated with AlexaFluor-conjugated second antibody overnight at 4°C. After washing in PBT, yolk sacs were flattened and mounted in aqueous mounting medium (LERNER LABORATORIES, Cat: 13800). Images were captured using a confocal microscope (Zeiss LSM 710).

### Histological analysis of embryos

After fixation, embryos were processed, embedded in paraffin, and sectioned at 8 µm. Hematoxylin and eosin staining was performed by standard staining protocol. For immunohistochemistry, sections were baked at 60°C for 1 hour, cleared in xylene, rehydrated through a descending concentration of ethanol for 2 minutes each ending in distilled water. Antigen retrieval was done in citrate buffer (0.01 M, PH = 6.0) using a pressure cooker for 30 minutes. Sections were cooled to room temperature and blocked with 5% goat serum diluted in PBS with 0.5% Triton-X-100 for 1 hour at room temperature. Sections were then incubated with primary antibody, SMA (1∶1000, SIGMA, Cat: A2547), and Pecam1 (1∶250, Santa Cruz, sc-1506-R) overnight at 4°C. After washing in PBT, sections were incubated with appropriate AlexaFluor–conjugated secondary antibody (1∶500 Invitrogen) for 1 hour at room temperature, counterstained with DAPI, and mounted in Vectashield mounting medium (Vector Laboratories, H-1400). Pictures were taken using a fluorescence microscope (OLYMPUS, 1X51).

### Cell culture and lentiviral expression of NICD2 and NICD3

Human aortic smooth muscle cells (HAoSMC) were purchased from Lonza and cultured in Dulbecco's Modified Eagle's Medium (DMEM) (Mediatech, Inc.) supplemented with 10% fetal bovine serum (FBS) (Hyclone), 2 mM glutamine, 1 mM sodium pyruvate and 100 U/ml penicillin-streptomycin. Cells between passages 6–9 were used for all experiments. For virus production, TN293 cells were purchased from Stratagene and cultured in 10% DMEM as above. All cultures were maintained in humidified 5% CO_2_ at 37°C. Human NOTCH2 intracellular domain (NICD2) cDNA (a gift from Dr. Igor Prudovsky) was cloned with a 3×-FLAG-tag attached to the 5′ end into pCDF1-MCS2-EF1-copGFP (System Biosciences). NOTCH3 intracellular domain (NICD3) with a 3×-FLAG-tag was made as described previously [14]. The lentivirus plasmids were transfected into TN-293 cells using Lipofectamine 2000 (Invitrogen), and the viral particles were amplified and purified as described [14]. Equal volumes of viral particles were diluted in 10% FBS in DMEM and were incubated with cells for 24 hours. The efficiency of infection was evaluated using GFP expression and qPCR. Viral particles were titrated to achieve 90% to 100% infection. Expression of cDNAs were confirmed using qPCR (not shown) and Western blot analysis using a FLAG antibody (SIGMA, F1804) (Figure 7).

### Statistical analysis

Data shown are representatives of at least three independent experiments and are presented as mean ± standard error of the mean (SEM). Data analyses were conducted using GraphPad Prism and comparisons of the data among 4 different groups were made using One-way ANOVA, followed by Newman-Keuls test. Differences were considered significant if *P*<0.05.

## Supporting Information

Figure S1 **Hematoxylin and eosin staining of transverse sections of E10.5 embryos through the descending aorta.** The overall structure of blood vessels appears relatively normal in the single mutant *Notch2^−/−^ (N2^−/−^;N3^+/+^), Notch3^−/−^ (N2^+/+^;N3^−/−^*) and double mutant, *Notch2^−/−^;Notch3^−/−^ (N2^−/−^;N3^−/−^)* embryos. 40× magnification.
(PDF)

Figure S2 **Quantification of Pecam1 and SMA staining of sectioned aortas.** Staining of Pecam1 and SMA was quantified by measuring the number of pixels with a set intensity and normalizing to vessel circumference. (A) At E10.5, the *Notch2^−/−^ (N2^−/−^;N3^+/+^)* and *Notch2^−/−^;Notch3^−/−^ (N2^−/−^;N3^−/−^)* embryos exhibit less SMA-positive staining intensity. (B) At E11.5, both Pecam1 and SMA expression is significantly reduced in the Notch2 and double mutant aortas. *Notch2^−/−^;Notch3^−/−^* embryos show an even greater loss of SMA expression compared to the *Notch2^−/−^* mice. *P*<0.05, * compared to wildtype control, # compared to *Notch2^−/−^*.
(PDF)

Figure S3 **Notch2 and Notch3 double mutant embryos have structurally normal hearts.** Transverse sections from wildtype and mutant embryos at E10.5 were stained for SMA (red) and Pecam1 (green) (A). All mutant embryos have normal SMA expression in the cardiomyocytes and Pecam1 staining of the endocardial cells. H&E staining of transverse sections through the outflow tract of wildtype and mutant embryos at E10.5 and E11.5 (B). At E10.5, hearts of the double mutant embryos are comparable to wildtype and single mutant embryos. A day later the Notch2/Notch3 double mutant embryo's outflow tract show signs of cellular atrophy, whereas the other three genotypes appear structurally normal. *Notch2^−/−^ (N2^−/−^;N3^+/+^), Notch3^−/−^ (N2^+/+^;N3^−/−^*) and double mutant, *Notch2^−/−^;Notch3^−/−^ (N2^−/−^;N3^−/−^)* embryos. 10× magnification.
(PDF)

Table S1 **Recovery of Notch2 (N2) and Notch3 (N3) mutant embryos at various gestational ages.**
(PDF)

Table S2 **Quantitative RT-PCR primer sequences.**
(PDF)
