# Supplementary material for: Notch2 and Notch3 Function Together to Regulate Vascular Smooth Muscle Development
Source: PLoS One. 2012 May 17;7(5):e37365. doi: 10.1371/journal.pone.0037365 (PMC3355134; doi:10.1371/journal.pone.0037365)
Supplement: Figure S1 — Hematoxylin and eosin staining of transverse sections of E10.5 embryos through the descending aorta. The overall structure of blood vessels appears relatively normal in the single mutant Notch2−/− (N2−/−;N3+/+), Notch3−/− (N2+/+;N3−/−) and double mutant, Notch2−/−;Notch3−/− (N2−/−;N3−/−) embryos. 40× magnification. (PDF) [file pone.0037365.s001.pdf]

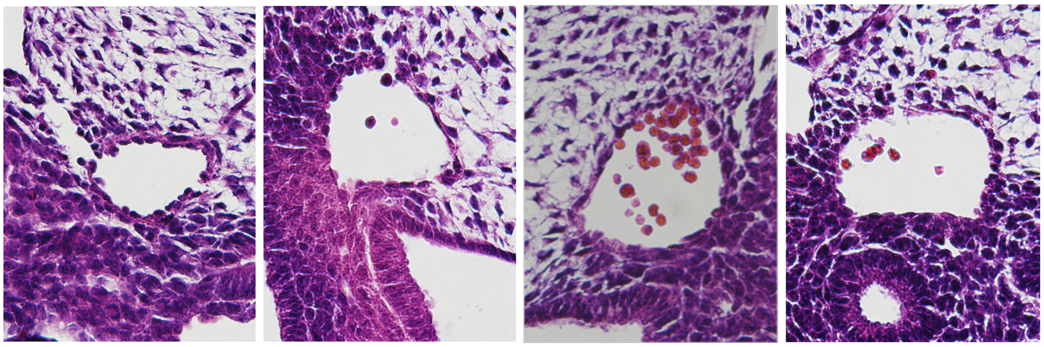

Figure S1. Hematoxylin and eosin staining of transverse sections of E10.5 embryos through the descending aorta. The overall structure of blood vessels appears relatively normal in the single mutant *Notch2*$^{-/-}$ *(N2*$^{-/-}$*;N3*$^{+/+}$*), Notch3*$^{-/-}$ *(N2*$^{+/+}$*;N3*$^{-/-}$) and double mutant, *Notch2*$^{-/-}$*;Notch3*$^{-/-}$ *(N2*$^{-/-}$*;N3*$^{-/-}$*)* embryos. 40X magnification.

Figure S1
