# Supplementary material for: Notch2 and Notch3 Function Together to Regulate Vascular Smooth Muscle Development
Source: PLoS One. 2012 May 17;7(5):e37365. doi: 10.1371/journal.pone.0037365 (PMC3355134; doi:10.1371/journal.pone.0037365)
Supplement: Figure S2 — Quantification of Pecam1 and SMA staining of sectioned aortas. Staining of Pecam1 and SMA was quantified by measuring the number of pixels with a set intensity and normalizing to vessel circumference. (A) At E10.5, the Notch2−/− (N2−/−;N3+/+) and Notch2−/−;Notch3−/− (N2−/−;N3−/−) embryos exhibit less SMA-positive staining intensity. (B) At E11.5, both Pecam1 and SMA expression is significantly reduced in the Notch2 and double mutant aortas. Notch2−/−;Notch3−/− embryos show an even greater loss of SMA expression compared to the Notch2−/− mice. P<0.05, * compared to wildtype control, # compared to Notch2−/−. (PDF) [file pone.0037365.s002.pdf]

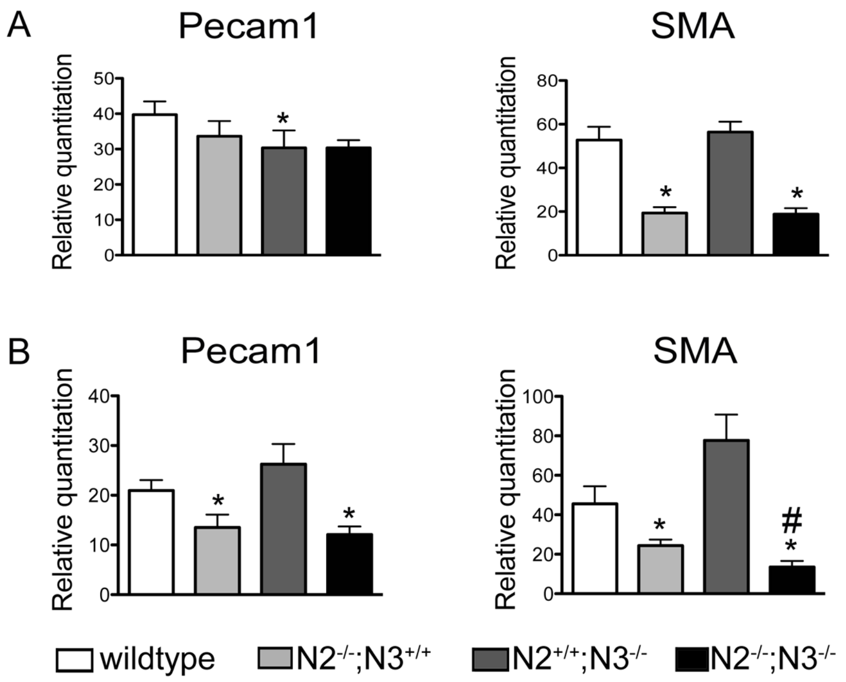


**Figure S2. Quantification of Pecam1 and SMA staining of sectioned aortas.** Staining of Pecam1 and SMA was quantified by measuring the number of pixels with a set intensity and normalizing to vessel circumference. (A) At E10.5, the *Notch2*$^{-/-}$ *(N2*$^{-/-}$*;N3*$^{+/+}$*)* and *Notch2*$^{-/-}$*;Notch3*$^{-/-}$ *(N2*$^{-/-}$*;N3*$^{-/-}$*)* embryos exhibit less SMA-positive staining intensity. (B) At E11.5, both Pecam1 and SMA expression is significantly reduced in the Notch2 and double mutant aortas. *Notch2*$^{-/-}$*;Notch3*$^{-/-}$ embryos show an even greater loss of SMA expression compared to the *Notch2*$^{-/-}$ mice. $P < 0.05$, * compared to wildtype control, # compared to *Notch2*$^{-/-}$.

Figure S2
