# Supplementary material for: Notch2 and Notch3 Function Together to Regulate Vascular Smooth Muscle Development
Source: PLoS One. 2012 May 17;7(5):e37365. doi: 10.1371/journal.pone.0037365 (PMC3355134; doi:10.1371/journal.pone.0037365)
Supplement: Figure S3 — Notch2 and Notch3 double mutant embryos have structurally normal hearts. Transverse sections from wildtype and mutant embryos at E10.5 were stained for SMA (red) and Pecam1 (green) (A). All mutant embryos have normal SMA expression in the cardiomyocytes and Pecam1 staining of the endocardial cells. H&E staining of transverse sections through the outflow tract of wildtype and mutant embryos at E10.5 and E11.5 (B). At E10.5, hearts of the double mutant embryos are comparable to wildtype and single mutant embryos. A day later the Notch2/Notch3 double mutant embryo's outflow tract show signs of cellular atrophy, whereas the other three genotypes appear structurally normal. Notch2−/− (N2−/−;N3+/+), Notch3−/− (N2+/+;N3−/−) and double mutant, Notch2−/−;Notch3−/− (N2−/−;N3−/−) embryos. 10× magnification. (PDF) [file pone.0037365.s003.pdf]

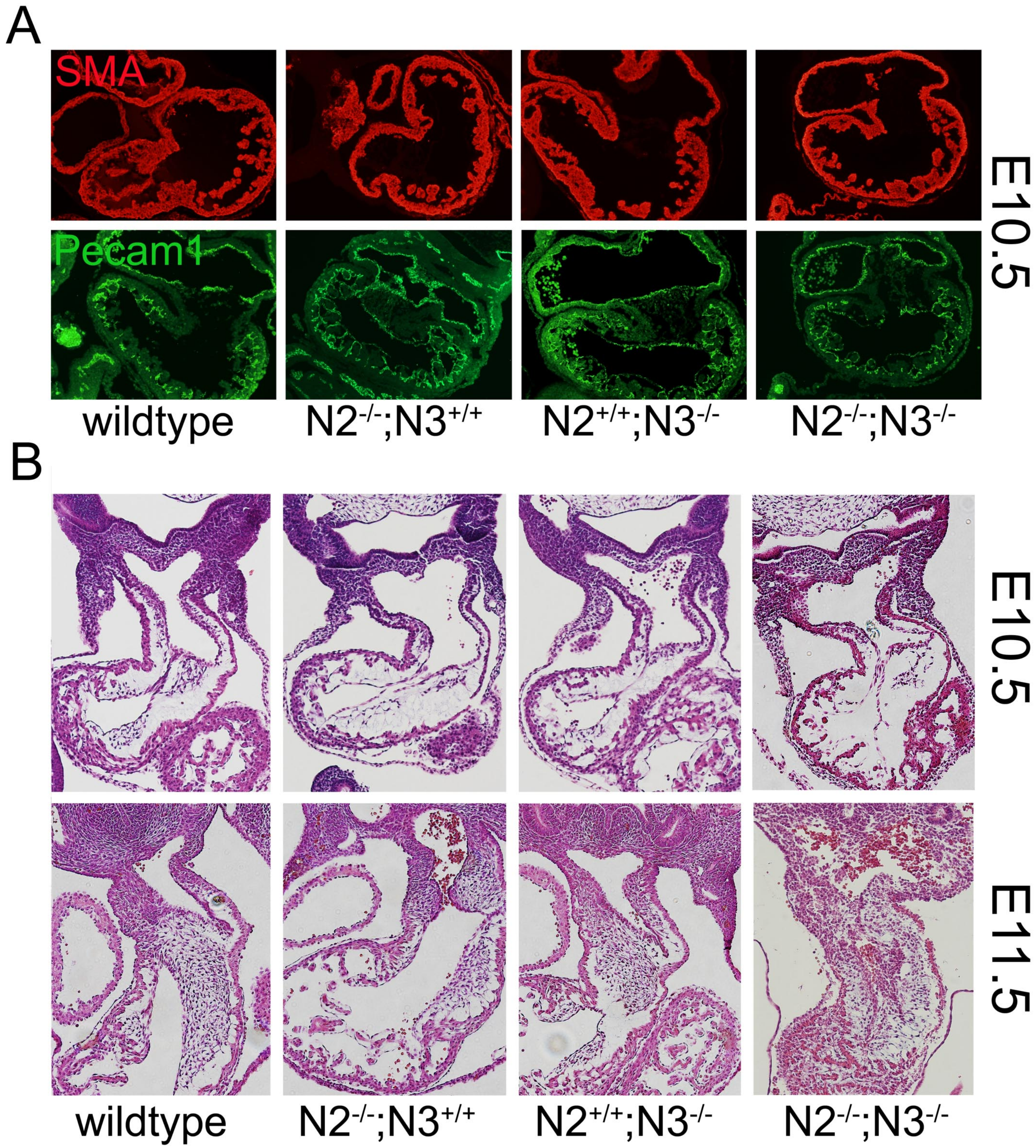
A
SMA
Pecam1
E10.5
wildtype
N2-/-;N3+/+
N2+/+;N3-/-
N2-/-;N3-/-
B
E10.5
E11.5
wildtype
N2-/-;N3+/+
N2+/+;N3-/-
N2-/-;N3-/-

Figure S3

Figure S3. Notch2 and Notch3 double mutant embryos have structurally normal hearts. Transverse sections from wildtype and mutant embryos at E10.5 were stained for SMA (red) and Pecam1 (green) (A). All mutant embryos have normal SMA expression in the cardiomyocytes and Pecam1 staining of the endocardial cells. H&E staining of transverse sections through the outflow tract of wildtype and mutant embryos at E10.5 and E11.5 (B). At E10.5, hearts of the double mutant embryos are comparable to wildtype and single mutant embryos. A day later the Notch2/Notch3 double mutant embryo's outflow tract show signs of cellular atrophy, whereas the other three genotypes appear structurally normal. *Notch2*$^{-/-}$ *(N2*$^{-/-}$*;N3*$^{+/+}$*), Notch3*$^{-/-}$ *(N2*$^{+/+}$*;N3*$^{-/-}$) and double mutant, *Notch2*$^{-/-}$*;Notch3*$^{-/-}$ *(N2*$^{-/-}$*;N3*$^{-/-}$*)* embryos. 10X magnification.
