# Supplementary material for: Notch2 and Notch3 Function Together to Regulate Vascular Smooth Muscle Development
Source: PLoS One. 2012 May 17;7(5):e37365. doi: 10.1371/journal.pone.0037365 (PMC3355134; doi:10.1371/journal.pone.0037365)
Supplement: Table S1 — Recovery of Notch2 (N2) and Notch3 (N3) mutant embryos at various gestational ages. (PDF) [file pone.0037365.s004.pdf]

Table S1. Recovery of Notch2 (N2) and Notch3 (N3) mutant embryos at various gestational ages

Age	Total Number	$N2^{+/+}$ $N3^{+/+}$	$N2^{-/+}$ $N3^{+/+}$	$N2^{-/-}$ $N3^{+/+}$	$N2^{+/+}$ $N3^{-/+}$	$N2^{-/+}$ $N3^{-/+}$	$N2^{-/-}$ $N3^{-/+}$	$N2^{+/+}$ $N3^{-/-}$	$N2^{-/+}$ $N3^{-/-}$	$N2^{-/-}$ $N3^{-/-}$
E10.5	286	14	39	17	34	75	34	18	33	22
	(Expected)	(18)	(36)	(18)	(36)	(72)	(36)	(18)	(36)	(18)
E11.5	249	16	39	19	36	64	24	13	25	13
	(Expected)	(12)	(24)	(12)	(24)	(48)	(24)	(12)	(24)	(12)
E12.5	119	6	19	5*	13	31	8	7	19	11*
	(Expected)	(7)	(15)	(7)	(15)	(30)	(15)	(7)	(15)	(7)

Embryos from the intercross of *$N2^{-/+}$;$N3^{-/+}$* mice were collected and genotyped at the indicated embryonic day (E) of gestation.

* At E12.5, 100% (11 out of 11) *$N2^{-/-}$;$N3^{-/-}$* embryos were being resorbed, compared to 40% (2 out of 5) of *$N2^{-/-}$; $N3^{+/+}$* embryos.
