# Supplementary material for: Notch2 and Notch3 Function Together to Regulate Vascular Smooth Muscle Development
Source: PLoS One. 2012 May 17;7(5):e37365. doi: 10.1371/journal.pone.0037365 (PMC3355134; doi:10.1371/journal.pone.0037365)
Supplement: Table S2 — Quantitative RT-PCR primer sequences. (PDF) [file pone.0037365.s005.pdf]

Table S2. Quantitative RT-PCR primer sequences

**Mouse Primers**	
SMA forward	TCCTGACGCTGAAGTATCCGATA
SMA reverse	GGTGCCAGATCTTTTCCATGTC
SM22α forward	CACAAACGACCAAGCCTTCTC
SM22α reverse	TCGGCTCATGCCGTAGGA
Cnn1 forward	AGCAGGAGCTGAGAGAGTGGAT
Cnn1 reverse	CCGTCTTTGAGGCCATACATG
Pecam1 forward	TGCGGTGGTTGTCATTGG
Pecam1 reverse	TGTTTGGCCTTGGCTTTCC
Heyl forward	CGCAGAGGGATCATAGAGAAACG
Heyl reverse	GCCAGGGCTCGGGCATCAAAGAA
Hey2 forward	CACATCAGAGTCAACCCCATGT
Hey2 reverse	GCCATGAGCAGAAGGCACTT
Hes1 forward	CCCCAGCCAGTGTCAACAC
Hes1 reverse	TGTGCTCAGAGGCCGTCTT
18S forward	GTTGGTTTTCGGAACTGAGGC
18S reverse	GTCGGCATCGTTTATGGTCG
GAPDH forward	GACGGCCGCATCTTCTTGT
GAPDH reverse	CACACCGACCTTCACCATTTT
**Human Primers**	
Notch2 forward	ACAGTTGTGTCTGCTCACCAGGAT (extracellular domain)
Notch2 reverse	GCGGAAACCATTCACACCGTTGAT (extracellular domain)
Notch3 forward	CCTAGACCTGGTGGACAAG (extracellular domain)
Notch3 reverse	ACACAGTCGTAGCGGTTG (extracellular domain)
SMA forward	CAAGTGATCACCATCGGAAATG
SMA reverse	GACTCCATCCCGATGAAGGA
Calponin forward	TGAAGCCCCACGACATTTTT
Calponin reverse	GGGTGGACTGCACCTGTGTA
SM-MHC forward	AGAAGCCAGGGAGAAGGAAACCAA
SM-MHC reverse	TGGAGCTGACCAGGTCTTCCATTT
Heyl forward	CATACAATGTCCTTGTGCAGTACACA
Heyl reverse	GCCAGGGCTCGGGCATCAAAGAA
Hey2 forward	TCGCCTCTCCACAACTTCAGA
Hey2 reverse	CATTCGGAGGGCTGAATCC
Hes1 forward	CGCGTGCAGTCCCAGATATA
Hes1 reverse	GCACCAGCTCCGGATCCT
18S forward	GTTGGTTTTCGGAACTGAGGC
18S reverse	GTCGGCATCGTTTATGGTCG
